# Improvement and Performance Evaluation of a Dual-Probe Heat Pulse Distributed Temperature Sensing Method Used for Soil Moisture Estimation

**DOI:** 10.3390/s22197592

**Published:** 2022-10-07

**Authors:** Jun-Cheng Yao, Bin Shi, Jie Liu, Meng-Ya Sun, Ke Fang, Jian Yao, Kai Gu, Wei Zhang, Ji-Wen Zhang

**Affiliations:** 1 School of Earth Sciences and Engineering, Nanjing University, Nanjing 210023, China; 2 School of Earth Sciences and Engineering, Hohai University, Nanjing 210098, China; 3 Jiangsu Province Engineering Investigation and Research Institute Co., Ltd., Yangzhou 225002, China; 4 China JK Institute of Engineering Investigations and Design Co., Ltd., Xi’an 710043, China

**Keywords:** soil moisture estimation, dual-probe heat pulse (DPHP), distributed temperature sensing (DTS), heating strategy, fitting algorithm

## Abstract

Large-scale measurements of soil moisture play a critical role in many fields, such as agriculture, hydrology, and engineering. The distributed temperature sensing (DTS) technology, based on a dual-probe heat pulse (DPHP), is a novel approach to realizing large-scale soil moisture estimation. However, the application of the method is limited by the complex optical cable layout, calculation algorithm, and lack of standardized heating strategy. In this paper, an improved DPHP-DTS method considering the soil bulk density was proposed. The measurement accuracy of the DPHP-DTS method under different heating strategies was studied in laboratory experiments, and its long-term stability in regard to the monitoring of soil moisture during natural evaporation in different soils was tested. The results show that the improved DPHP-DTS method can accurately measure the soil moisture, and the fitting algorithm can reduce the error caused by the accuracy of the DTS temperature measurement under the low-power heating strategy. Its measurement accuracy increases with the increase in the heating strength and duration. In addition, the improved DPHP-DTS method can describe soil evaporation in both sand and loess with good reliability and stability.

## 1. Introduction

The soil moisture content is an important factor in environmental ecology, engineering geology, and agriculture engineering. It is necessary to accurately estimate the in situ distribution of the soil moisture. There are various monitoring methods for the soil moisture content on different spatial scales [1,2,3,4,5]. Distributed temperature sensing technology (DTS) has the advantages of a high sensitivity, anti-electromagnetic interference, anti-corrosion, a good durability, potentially low cost, and a capacity for long-distance distributed measurements, which can measure the soil moisture content on intermediate spatial scales ranging from meters to kilometers [6,7,8]. At present, the actively heated distributed temperature sensing (AH-DTS) technology used for soil moisture monitoring includes two approaches: a single-probe heat pulse distributed temperature sensing (SPHP-DTS) method and a dual-probe heat pulse distributed temperature sensing (DPHP-DTS) method [9,10].

The SPHP-DTS method heats an AH-DTS cable and calculates the soil moisture content through the temperature curve measured by the AH-DTS cable itself. The SPHP-DTS method can enable the continuous long-term monitoring of the soil moisture in the direction of the AH-DTS cable. Its calculation method includes the cumulative temperature (*T_cum_*) method, the temperature characteristic value (*T_t_*) method, and the soil thermal conductivity (*λ*) method. However, these methods require complex calibration models, without a nonlinear functional relationship between *T_cum_*/*T_t_*/*λ* and *θ* [11]. Thus, soil-specific calibrations are necessary [12,13]. In addition, the SPHP-DTS method requires a long heating duration of about 20 min and consumes a large amount of energy.

Due to the abovementioned limitations of the SPHP-DTS method, Benítez-Buelga et al. proposed a new DPHP-DTS method based on the principle of a dual-probe heat pulse in 2014 [14,15,16]. It used two AH-DTS cables in parallel, which can measure the soil volumetric heat capacity (*C_v_*) and thermal diffusivity (*k*) by heating one of the AH-DTS cables and measuring the thermal response of the other cable. Since there is a linear relationship between *C_v_* and *θ*, the soil moisture content (*θ*) can be calculated [17]. Compared with the SPHP-DTS method, the DPHP-DTS method has the advantages of a short heating duration and lack of need for special calibration before monitoring [18].

However, the use of the DPHP-DTS method still has some limitations. Compared with the traditional DPHP method, due to the larger diameter and spacing of the AH-DTS cables, the maximum temperature rise value measured by the temperature cable is lower, and the measurement result is easily affected by the structure of the AH-DTS cables. Therefore, Shehata et al. estimated the soil volumetric heat capacity (*C_v_*, J m^−3^ K^−1^) and soil moisture content (*θ*, m^3^ m^−3^) by fitting the temperature heating curve [18]. They found that using an appropriate mathematical model to fit the whole heat pulse signal was far more reliable than the calculation method, relying on individual temperature measurements. However, the fitting algorithm used by Shehata et al. had certain limitations during in situ testing, since its AH-DTS cable layout and heating methods were relatively complicated, with two AH-DTS cables alternately releasing heat pulses and four temperature-measuring AH-DTS cables [18]. Complex layouts and heating methods are not conducive to in situ application. In addition, the heating strength used in the DPHP-DTS method is relatively high, generally 30–40 W m^−1^. In field monitoring, if the resistance of the heating cable is small, the current may be too large to reduce the durability of the inner heating cable; however, if the resistance of the heating cable is too large, the test distance will be limited [19]. The selection of an appropriate heating strategy has become a key factor in the in situ application of the DPHP-DTS method. Additionally, the DPHP-DTS method only takes sand as the research object [14,18,20,21], and the contact impedances between different types of soil and sensors are different. Hence, the performance of the DPHP-DTS method in relation to different soils under different heating strategies requires further evaluation. Moreover, variations in the soil bulk density can influence the soil moisture measurement [11], but this is not considered in the DPHP-DTS method.

This paper describes how we improved the DPHP-DTS method by using a new fitting algorithm, considering the bulk density variation. The performance of the improved DPHP-DTS method under different heating strategies and in different soils was evaluated. Finally, the method was used for monitoring the temporal and spatial distribution of the water content during the evaporation process so as to test its long-term stability. This study provided technical support for the in situ application of the DPHP-DTS method.

## 2. Theory

### 2.1. DTS Technology

The DTS is a temperature sensor based on the Raman scattering effect, which is used to measure temperature and based on the time domain reflection technique of light. The temperature measurement principle assumes that the energy of pulsed pump light injected into a fiber optic produces two beams of backscattered Raman light, of which the Stokes scattered light, with a wavelength greater than the incident light, is not affected by temperature, while the anti-Stokes scattered light, with a wavelength smaller than the incident light, has a strong temperature dependence. As a result, the temperature can be calculated from the ratio between the Stokes and anti-Stokes intensities along the length of the fiber optic. The temperature at a given point in the fiber optic can be estimated by [22,23,24,25]:

(1) $$T\left(z\right)=\frac{\Delta E/k}{\ln C-\ln R\left(z\right)+\Delta \alpha z},$$

where *T*(*z*) is the temperature (K) at a distance *z* (m) along the fiber optic; ∆*E* represents the difference between the molecular energy states driving the Raman scattering (J); *k* is the Boltzmann constant (J K^−1^); *R*(*z*) is the ratio between the Stokes and anti-Stokes intensities; ∆α is the differential attenuation of the backscattered Stokes and anti-Stokes intensities; and *C* is a calibratable parameter, which is related to the wavelength and frequency of the incident light, the backscattered Raman light, the instrument’s photon detector, and the operating conditions of the DTS instrument.

### 2.2. ICPC Model for the DPHP-DTS Method

**Figure 1:**
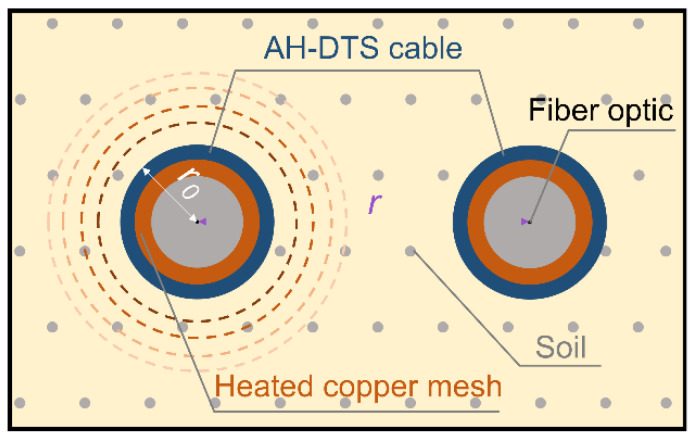
Schematic diagram of the DPHP-DTS method.

Figure 1 is a schematic diagram of the DPHP-DTS method. There are two AH-DTS optical cables with a radius *r*_0_ and an interval distance *r* lying in parallel. The left AH-DTS cable acts as a heating probe to generate heat pulses. The right AH-DTS cable acts as a temperature probe to measure the temperature. 

According to an alternative semi-analytical model proposed by Knight et al. [26] for identical cylindrical perfect conductors (ICPC), the analytical solution for the temperature (*T*) measured by the temperature probe during continuous heating is as follows:

(2) $$\mu =\sqrt{pC_{v}/\lambda },$$

(3) $$f\left(p,r_{0},C_{v0},C_{v}\right)=1/\left\{\mu r_{0}\left[K_{1}\left(\mu r_{0}\right)+\left(\frac{\mu r_{0}C_{v0}}{2C_{v}}\right)K_{0}\left(\mu r_{0}\right)\right]\right\},$$

(4) $$T_{r}\left(p\right)=f^{2}\left(p,r_{0},C_{v0},C_{v}\right)\frac{qK_{0}\left(\mu r\right)}{2\pi \lambda p},$$

where *T_r_*(*p*) is the Laplace transform of *T_r_*(*t*), measured by the temperature probe. The Laplace domain solution for *T_r_*(*t*) is obtained using the definition:

(5) $$T_{r}\left(p\right)=\int_{0}^{\infty }T_{r}\left(t\right)\exp\left(-pt\right)dt,$$

where *p* is the Laplace transformation variable; *t* is the time (s); *q* is the heating strength (W m^−1^) per unit length of the heating probe; *r* is the distance (m) between the heating probe and the temperature probe; *T_r_*(*t*) is the temperature (K) measured by the temperature probe; *r*_0_ is the radius of the probe (m); *λ* is the soil thermal conductivity (W m^−1^ K^−1^); *C_v_* is the soil volumetric heat capacity (J m^−3^ K^−1^); *C_v_*_0_ is the volumetric heat capacity (J m^−3^ K^−1^) of the probe; *K*_0_ is the second kind of 0-order modified Bessel function; and *K*_1_ is the second kind of 1-order modified Bessel function.

### 2.3. Principle of the Soil Moisture Calculation 

In the DPHP-DTS method, the soil moisture can be calculated from its linear relationship with the soil volumetric heat capacity. The soil volumetric heat capacity (*C_v_*) is expressed as the weighted sum of the heat capacities of the soil constituents. Since the specific heat capacity of air is much smaller, it can be ignored. Therefore, the *C_v_* can be estimated by [27]:

(6) $$C_{v}=\rho_{w}c_{w}\theta_{v}+\rho_{b}c_{s},$$

where *θ_v_* is the volumetric water content (m^3^ m^−3^); *ρ_w_* is water density (g cm^−3^); *c_w_* is the specific heat capacity of the water, equal to 4180 J kg^−1^ K^−1^; *c_s_* is the specific heat capacity of the soil solid (J kg^−1^ K^−1^); and *ρ_b_* is the soil bulk density (g cm^−3^).

Therefore, the soil volumetric water content (*θ_v_*) can be calculated from the *C_v_* obtained by the DPHP-DTS method:

(7) $$\theta_{v}=\frac{C_{v}-\rho_{b}c_{s}}{\rho_{w}c_{w}}.$$

## 3. Improvement of the DPHP-DTS Method

### 3.1. Fitting Algorithm 

Since the expression of *T_r_*(*p*) in the ICPC model is relatively complex, the exact expression of *T_r_*(*t*) cannot be solved. In the fitting algorithm, it is necessary to perform the Laplace numerical inversion of *T_r_*(*p*) to obtain the numerical solution of *T_r_*(*t*) [28]. Finally, under the heat pulse with the heating duration *t*_0_, the theoretical temperature *T*(*t*) of the temperature probe is expressed as:

(8) $$T\left(t\right)=\left\{\begin{array}{l} T_{r}\left(t\right)\;\;\;\;\;\;;\;\left(0<t\leq t_{0}\right)\\ T_{r}\left(t\right)-T_{r}\left(t-t_{0}\right)\;\;;\;\;\left(t>t_{0}\right) \end{array}\right..$$

In the fitting algorithm and accuracy evaluation, the root mean square error (RMSE) is an important metric, and it is expressed as:

(9) $$\mathrm{RMSE}=\sqrt{\frac{1}{n}\sum_{i=1}^{n}{\left(y_{pre,i}-y_{mea,i}\right)}^{2}},$$

where *y_pre,i_* is the predicted value; *y_mea,i_* is the measured value; and *n* is the number of data points. The RMSE is in the same unit as the measured value. When calculating the temperature root mean square error (*RMSE_tem_*), *y_pre,i_* is the predicted value obtained by the Laplace numerical inversion (K); *y_mea,i_* is the temperature measured by the temperature probe (K); and the unit of *RMSE_tem_* is also K. In the evaluation of the volumetric specific heat capacity and moisture content, the *y_pre,i_* and *y_mea,i_* in Equation (9) represent the results obtained by the oven drying method and the DPHP-DTS method, respectively. The volumetric specific heat capacity root mean square error (*RMSE_cap_*) and moisture root mean square error (*RMSE_moi_*) indicate the difference between the oven drying method and the DPHP-DTS method, which does not represent the absolute error of the DPHP-DTS method [29].

**Figure 2:**
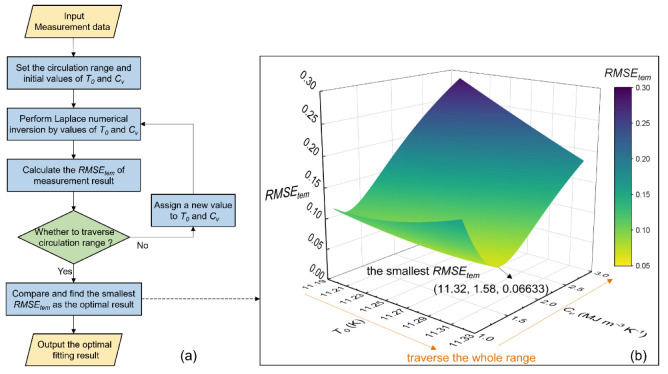
Principle of the fitting algorithm: (**a**) flow chart of the fitting algorithm and (**b**) minimization process of *RMSE_tem_*.

The flow chart of the fitting algorithm is shown in Figure 2a. First, the temperature data (*y_mea,i_*) measured by the right AH-DTS cable is input. After setting the initial circulation range of the parameter *C_v_* (1~3 MJ m^−3^ K^−1^) and an initial value of *C_v_*, the Laplace numerical inversion is performed to obtain the predicted value (*y_pre,i_*) of the *T*(*t*) numerical solution, which can calculate the *RMSE_tem_* between *y_pre,i_* and *y_mea,i_*. Then, the *C_v_* is re-assigned, and the above calculation is performed again until the whole range is traversed. Finally, the *C_v_* value with the smallest *RMSE_tem_* in the entire range is defined as the optimal fitting result, and the *C_v_* value corresponding to the optimal fitting result is output. Figure 2b is an example showing the minimization process of *RMSE_tem_*.

At the beginning of the test, it was found that the temperature fluctuated to a certain extent due to the temperature measurement accuracy of the DTS. The different values of the initial temperature (*T*_0_) had a great influence on the final fitting result. Since the theoretical temperature value of the first 30 s is almost 0, in the subsequent algorithm optimization, the first four measured temperature values (t = 0, 10, 20, 30 s) are selected as the circulation range of *T*_0_. All possible *T*_0_ values in the circulation range are traversed with a circulation step size of 0.01 K. This adjustment can reduce the influence of *T*_0_ on the fitting result and make the fitting result more accurate.

### 3.2. Bulk Density Calibration Method 

During in situ monitoring, it is difficult to measure the soil bulk density at different depths with the cutting ring method, while the soil mass water content is easier to be measured by the oven drying method. The relationship between the mass water content (*ω_G_*), bulk density (*ρ_b_*), and volumetric water content (*θ_v_*) is as follows:

(10) $$\theta_{v}=\frac{\rho_{b}}{\rho_{w}}\omega_{G},$$

where *ω_G_* is the mass water content, kg kg^−1^. Then, substitute Equation (10) into Equation (6) to obtain the following equation:

(11) $$C_{v}=\rho_{b}\left(c_{w}\omega_{G}+c_{s}\right),$$

where only *ρ_b_* is an unknown parameter. There is a linear relationship between *C_v_* and *ω_G_*. The slope obtained by linear fitting is the calibration value of *ρ_b_*.

Assuming that *ρ_b_* does not change over a certain period, the *C_v_* and *ω_G_* are measured by the DPHP-DTS method and oven drying method, respectively, under different water content conditions in this period. The *ρ_b_* of this period is estimated by fitting these two parameters. Then, the above steps are repeated in another period. The different periods can be compared to determine whether *ρ_b_* has changed. This method can reduce the measurement error induced by the bulk density variation.

In general, the improved DPHP-DTS method uses the ICPC semi-analytical model to optimally fit the temperature *T*(*t*) measured by the temperature probe, which can provide the optimal fitting result *C_v_*. Then, the calibration value of *ρ_b_* is obtained by the linear fitting of the *C_v_* and *ω_G_*. Finally, *θ_v_* can be calculated by Equation (7).

## 4. Materials and Method

### 4.1. Experimental Setup

**Figure 3:**
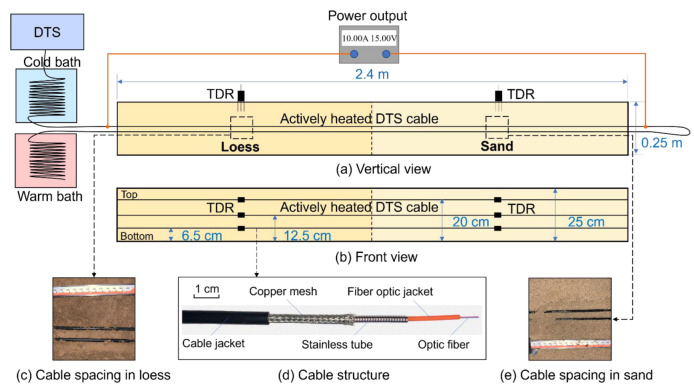
Schematic of the experimental setup: (**a**) the vertical perspective of the model box, (**b**) the front perspective of the model box, (**c**) the photograph of the cable spacing in loess, (**d**) the photograph of the cable structure, and (**e**) the photograph of the cable spacing in sand.

To evaluate the performance of the improved DPHP-DTS method under different heating strategies in different soils, a laboratory test was carried out. The schematic of the experimental setup is shown in Figure 3. Firstly, the soil samples were dried in an oven at 105 °C, and then water was sprayed onto the dry soil to prepare the sand and loess samples. Furthermore, the sand and loess samples were mixed fully with water, respectively, and sealed in plastic bags for 24 h to ensure the uniform distribution of the water. Next, the prepared loess and sand samples were filled in layers into an acrylic model box with a length, width, and height of 2.4 m × 0.25 m × 0.25 m, respectively. The initial characteristics of the soil sample are shown in Table 1.

At the same time, the AH-DTS cables are laid in the model box in three layers. The heights of the three layers of the AH-DTS cables from the bottom of the model box are 6.5 cm, 12.5 cm, and 20 cm, respectively (see Figure 3b). The AH-DTS cables in each layer are arranged in a U-shaped layout (See Figure 3a). Since the accurate distancing between the AH-DTS cables has a great influence on the test results, two AH-DTS cables at the same height are fixed with clips to ensure that the same distance is maintained. The AH-DTS cable responsible for heating is connected to the power output through the electric wire. In addition, a calibrated time domain reflectometry (TDR) moisture sensor is installed in the center of each layer of the sand and loess for the comparative monitoring of changes in the water content (Figure 3a,b).

The actual distance between the two AH-DTS optical cables was photographed vertically with a high level of precision (Figure 3c,e) and measured using Photoshop software. The distances *r* between the AH-DTS cables at a height of 12.5 cm in the loess and sand are both 16.90 mm. The cable diameter is 5.25 mm. The structure of the AH-DTS cable is shown in Figure 3d. According to the cable structure, referring to the calculation equation of the material’s specific heat capacity by Basinger et al. [30], the AH-DTS cable volumetric heat capacity (*C_v_*_0_) was calculated as 2.43 MJ m^−3^ K^−1^.

### 4.2. Heating Strategies and Measuring Protocol

The characteristics of the soil used during the testing of the different heating strategies are shown in Table 1. The soil surface was covered with plastic film during each heating period to avoid possible evaporation. The heating cable was heated according to the heating strategy in Table 2. Here, L, M, and H, respectively, represent the low, medium, and high heating strengths, and the numbers after the letters indicate the length of the heating duration.

During the evaporation test, the plastic film on the top of the model box was uncovered to ensure the soil’s natural evaporation state. From 27 January to 6 July, the soil samples with different water contents were measured in the range of 0~0.2 kg kg^−1^. During the evaporation process, soil samples were taken at different times in order to obtain the corresponding mass water content using the oven drying method.

The temperature measurement accuracy of DTS (ULTIMA^TM^ DTS) is ±0.1 K, the spatial resolution is 0.6 m, and the sampling interval is 10 s. The final temperature measurement results are calibrated using a double-ended water bath. Each measurement is separated by at least 2 h to avoid heat accumulation.

The calibration value of *ρ_b_* at the corresponding position can be obtained by fitting *C_v_* and *ω_G_* in Equation (11) at different times, and then *θ_v_*, measured by the DPHP-DTS method, can be calculated using Equation (7). Moreover, the *C_s_* was determined by the differential scanning calorimetry (DSC) method in advance, giving 680 J kg^−1^ K^−1^ for sand and 750 J kg^−1^ K^−1^ for loess. Hence, the *C_v_* can be calculated according to Equation (6), using the *θ_v_* and *ρ_b_*. In addition, the *λ* was determined by the steady-state plate method (HC-110, EKO, Japan), configuring the soil samples with the same *θ_v_* and *ρ_b_*.

## 5. Results and Discussion

### 5.1. Determination of Cv under Different Heating Strategies 

**Figure 4:**
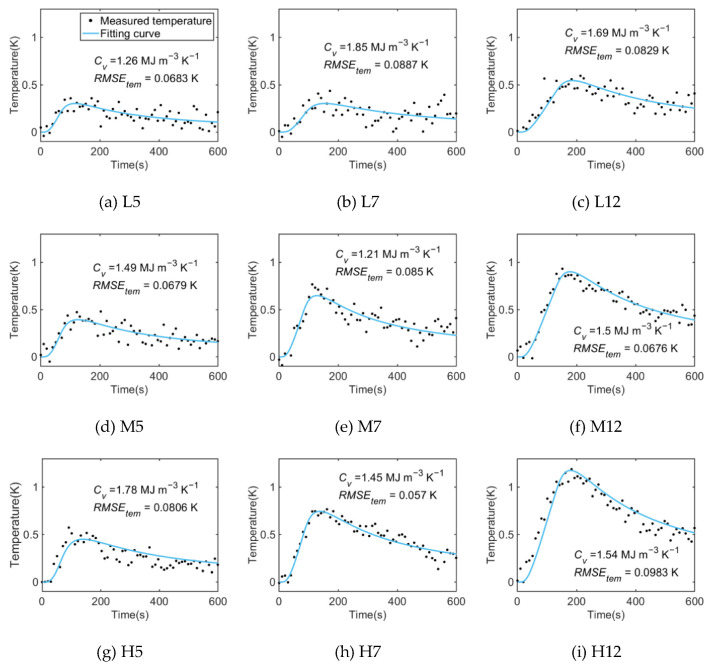
Fitting curves of the sand heat pulse process under different heating strategies.

The soil volumetric heat capacity (*C_v_*) is obtained by fitting the thermal pulse temperature change process in the sand and loess under different heating strategies using the improved algorithm. Figure 4 shows the fitting curves of the sand.

At the beginning of the heating, the temperature rises rapidly, with a strong fitting effect under all the heating strategies. Meanwhile, in the fitting curves of the short heating period (Figure 4a,d,g), it can be seen that the fitting error of the temperature drop section is large because the maximum temperature rise value (Δ*T_max_*) is small and the temperature drop section is longer, resulting in a poor fitting effect. Such a problem still exists under the heating strategy of L7 (Figure 4b). However, as the heating duration increases, the fitting effect improves and the measurement results become more accurate under the heating strategy of L12 (Figure 4c).

In addition, the *RMSE_tem_* values under all the heating strategies selected in this test were less than 0.1 K, and the DTS temperature measurement accuracy is 0.1 K. This indicates that the temperature fluctuation is within the scope of the measurement accuracy of the instrument, which proves the feasibility of the DPHP-DTS method under the low-power heating strategy.

**Figure 5:**
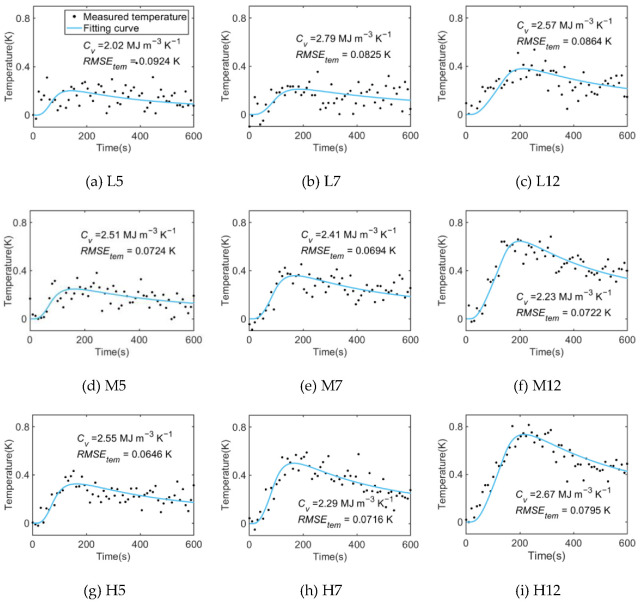
Fitting curves of the loess heat pulse process under different heating strategies.

Similarly, the thermal pulse of the loess under different heating strategies is fitted by the same method, and the fitting results are shown in Figure 5. The temperature change trend and fitting results of the loess in the thermal pulse process under different heating strategies are consistent with those of sand. Due to the different physical properties, such as the water content, specific heat capacity, and thermal conductivity, under the same heating strategy, the Δ*T_max_* of the loess is slightly lower than that of sand. In addition, the *RMSE_tem_* value of the loess is also less than 0.1 K, which proves that the fitting algorithm is applicable to both loess and sand.

Figure 4a and Figure 5a are, respectively, the fitting results of the sand and loess under the heating strategy of L5, with the smallest heating strength and the shortest heating duration in the whole test. Under this strategy, the Δ*T_max_* is only about 0.3 K, resulting in a poor fitting effect under the DTS temperature measurement accuracy of 0.1 K. In contrast, under the heating strategy of H12 (Figure 4i and Figure 5i), the Δ*T_max_* increases significantly, and the fitting effect and measurement accuracy of *C_v_* in the loess and sand are better. The influence of Δ*T_max_* on the fitting effect and the measurement accuracy of *C_v_* shows a positive correlation trend. Therefore, under a low heating strength (20 W m^−1^), the heating duration can be appropriately increased so as to increase the maximum temperature rise value, as indicated by the fitting results of the sand and loess under the heating strategy of L12 (Figure 4c and Figure 5c), where the fitting effect is significantly improved compared to the heating strategy of L5.

**Figure 6:**
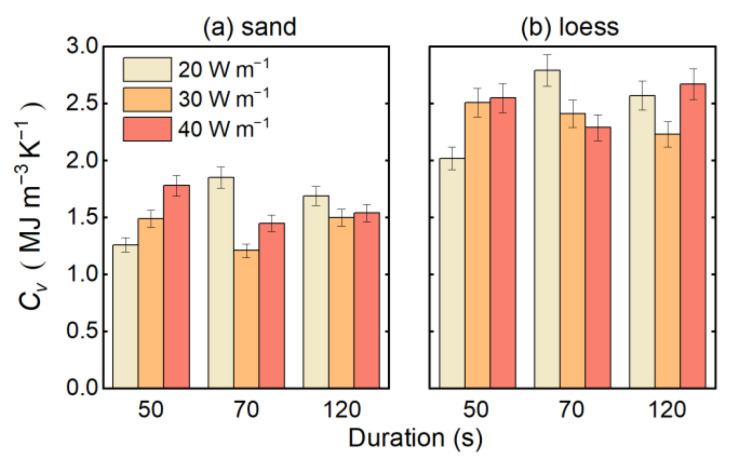
Measurement results of the *C_v_* of the sand (**a**) and loess (**b**) under different heating strategies.

Figure 6 shows the measurement results of the *C_v_* of the two soils under different heating strategies. Substituting the parameters obtained using the traditional measurement methods in Table 1 into Equation (6), the real *C_v_* of the sand and loess can be calculated as 1.5553 MJ m^−3^ K^−1^ and 2.5231 MJ m^−3^ K^−1^, respectively. Under all the heating strategies, the *RMSE_cap_* of the sand and loess are 0.2056 MJ m^−3^ K^−1^ and 0.2362 MJ m^e^ K^−1^, respectively. The accuracy of the measurement of the *C_v_* by the DPHP-DTS method increases with the increase in the heating duration. When the heating duration is 120 s, the *RMSE_cap_* of the loess is the smallest, which is 0.1912 MJ m^−3^ K^−1^. When the heating duration is 70 s and 50 s, the *RMSE_cap_* of the loess are 0.2148 MJ m^−3^ K^−1^ and 0.2910 MJ m^−3^ K^−1^, respectively. The change in the trend of the sand *C_v_* measurement accuracy with the heating duration is the same as that of loess. Similarly, when the heating strength is 40 W m^−1^, the *RMSE_cap_* of the sand is the smallest, at 0.1436 MJ m^−3^ K^−1^. When the heating strength is 30 W m^−1^ and 20 W m^−1^, the *RMSE_cap_* of the sand is 0.2053 MJ m^−3^ K^−1^ and 0.2531 MJ m^−3^ K^−1^, respectively. As the heating strength and heating duration increase, the measurement accuracy can be improved. Therefore, in the application of the method, the Δ*T_max_* of DPHP-DTS should be estimated according to the physical properties of the soil in advance. According to the soil types and soil moisture conditions, a suitable heating strategy can be used to improve the measurement accuracy.

In conclusion, the improved DPHP-DTS method can be applied to different soil types, and there is a positive correlation between the Δ*T_max_* value and the measurement accuracy of *C_v_*. Under the low-power heating strategy, the DPHP-DTS method can also achieve accurate measurements by appropriately prolonging the heating duration.

### 5.2. Bulk Density Calibration Results

**Figure 7:**
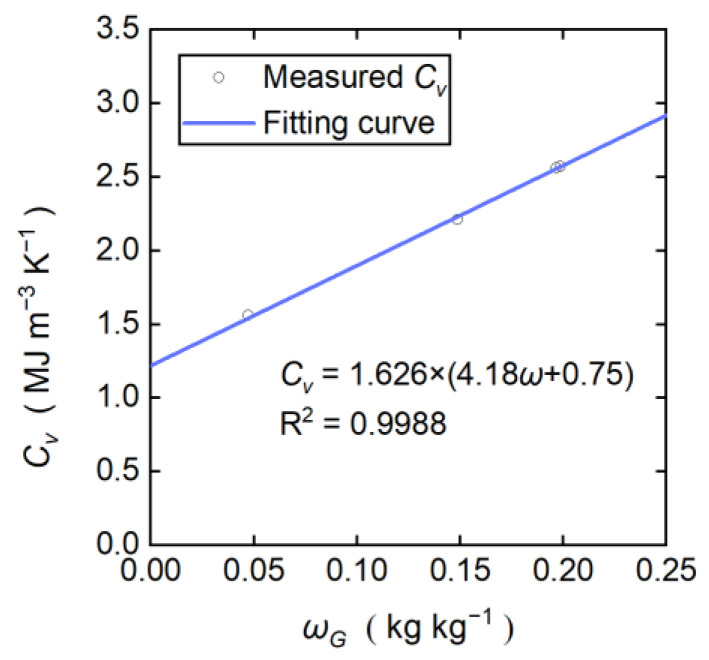
Fitting curve of the loess *ρ_b_* at a height of 12.5 cm.

During the evaporation of the water in sand and loess, the *ω_G_* gradually decreased, but the *ρ_b_* did not change. Therefore, the *ρ_b_* at different locations can be obtained by fitting the *C_v_* and *ω_G_* obtained by the DPHP-DTS method and the oven drying method, respectively, in Equation (11). Figure 7 is the fitting curve of the loess at a height of 12.5 cm. Its coefficient of determination (R^2^) is 0.9988, which is close to 1, with a good fitting result. The percentage error of the results compared with the *ρ_b_* using the cutting ring method is less than 3%. The good fitting effect proved that the *ρ_b_* did not change during the laboratory test.

Table 3 shows the fitting results of the *ρ_b_* of the loess and sand at different heights. In the actual backfilling process during field application, it is difficult to measure *ρ_b_* by taking the soil cutting ring samples at different depths, but it is easier to measure *ω_G_* using the soil sampler and oven. This test found that a relatively accurate *ρ_b_* can be estimated by measuring just a few different actual *ω_G_* values. Therefore, the improved DPHP-DTS method is suitable for the calibration of *ρ_b_* during in situ monitoring.

### 5.3. Determination of the Soil Moisture

**Figure 8:**
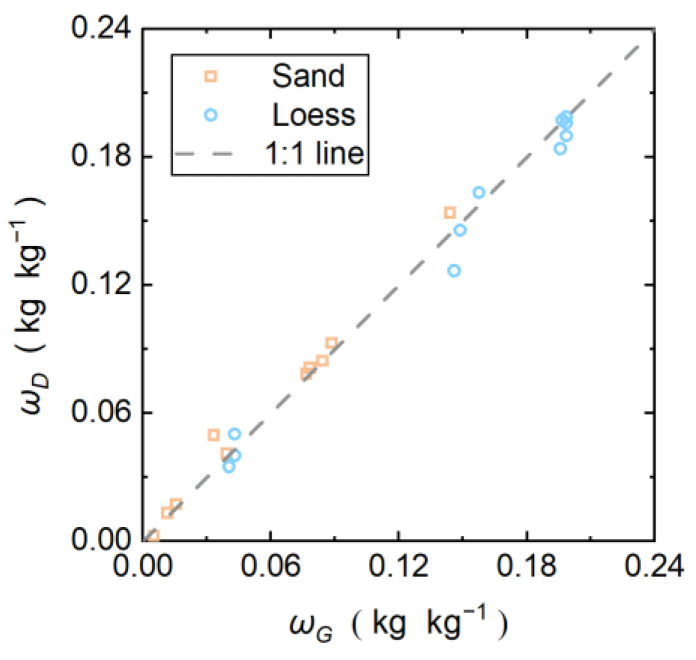
Comparison of the soil moisture in sand and loess estimated by DPHP-DTS method (*ω_D_*) and oven drying method (*ω_G_*).

Figure 8 shows the measurement results of *ω_G_* estimated by the DPHP-DTS method. It can be seen that the soil moisture error increases with the soil moisture content, which is consistent with previous studies [6,9,31]. There are two main reasons for this. Firstly, soil with a high water content has a lower Δ*T_max_*, so that the relative error of the heating curve is larger than that of soil with a low water content under the same DTS temperature measurement accuracy. Secondly, when the water content is close to or exceeds the plastic limit of the soil, the free water content is high, and the increase in the temperature of the AH-DTS cable may cause water vapor migration, resulting in a measurement error.

Using Equation (9), the *RMSE_moi_* between the water content results measured by the DPHP-DTS method and the water content measured by the oven drying method is calculated. The *RMSE_moi_* of sand is 0.006425 kg kg^−1^, and the *RMSE_moi_* of loess is 0.008127 kg kg^−1^. In comparison, the moisture content test results of the sand are more accurate, which is related to the higher Δ*T_max_* value of the sand. The *RMSE_moi_* values of both soils are less than 0.01 kg kg^−1^. Compared with the oven drying method, the improved DPHP-DTS method is reliable and applicable to both sand and loess.

### 5.4. Application in Soil Evaporation

**Figure 9:**
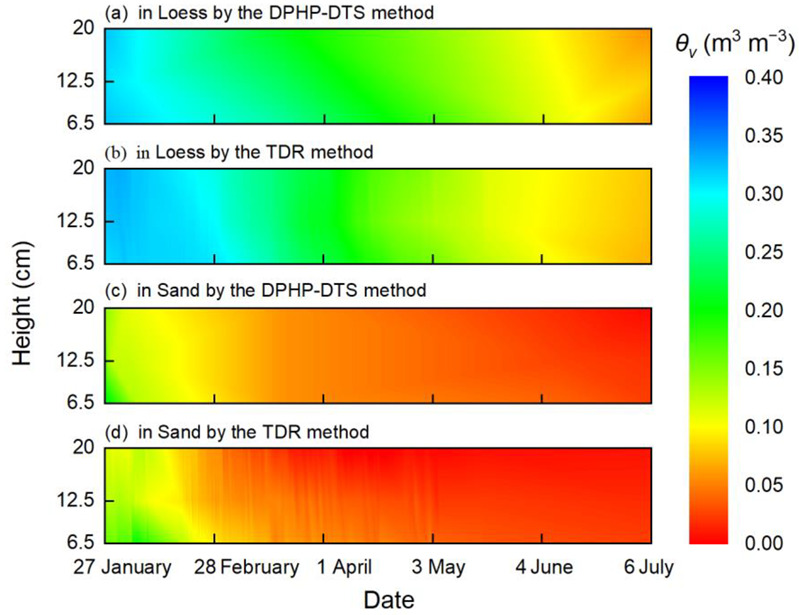
Temporal and spatial distribution of *θ_v_* during evaporation from 27 January to 6 July: (**a**) *θ_v_* in loess measured by the DPHP-DTS method, (**b**) *θ_v_* in loess measured by the TDR method, (**c**) *θ_v_* in the sand measured by the DPHP-DTS method, and (**d**) *θ_v_* in sand measured by the TDR method.

Using the DPHP-DTS method, the temporal and spatial distribution diagrams of the *θ_v_* of the sand and loess during evaporation can be drawn (Figure 9). The results are consistent with the TDR method. The evaporation of the water in sand mainly occurs from January to March (Figure 9c,d), while the evaporation in loess lasts until July (Figure 9a,b). In the depth direction, the *θ_v_* of the topsoil is lower than that of the subsoil, and the evaporation plane tends to move downwards gradually. This is because the topsoil *θ_v_* gradually decreases, and the hydraulic conductivity and soil suction gradually decrease in the evaporation process. The capacity of the water supply from the subsoil to the topsoil also weakens, causing the change in the subsoil *θ_v_* to be slow.

The evaporation rates of the sand and loess are different. At the beginning of the evaporation process, the evaporation rate of the sand is faster than that of the loess. From 27 January to 28 February, the evaporation rate of the loess is 0.39 mm d^−1^, and the evaporation rate of the sand is 0.55 mm d^−1^. However, as the evaporation process continues, the evaporation rate of the loess is faster than that of the sand. From 27 January to 6 July, the evaporation rate of the loess is 0.39 mm d^−1^, and the evaporation rate of the sand is 0.22 mm d^−1^. Overall, in contrast to the uniform evaporation rate of the loess, the evaporation rate of the sand changes from fast to slow. Because the pore size of the sand is large, the water retention capacity in the early evaporation stage generally depends on the contact between the sand particles, with the water in the sand pores easily flowing between the pores. Following the first stage of constant-rate soil drying, the evaporation rate starts to decline as the moisture content of the soil surface decreases, and the evaporation is mainly determined by the soil water holding capacity in the residual stage [32]. During the residual stage of evaporation, the water transfer capacity of the sand is weak, and the evaporation loss on the surface cannot be replenished in time, resulting in a slow evaporation rate of the sand. However, the loess always remains in the constant-rate evaporation stage, because the pore size of loess is small, easily forming a relatively stable water transport channel. At the same time, the liquid water is slowly and continuously supplied to the evaporation surface under the action of the capillary force, thus resulting in a uniform evaporation rate of the loess.

Comparing the dynamic monitoring results obtained by the DPHP-DTS method and TDR method, the trend of the variation in the water content measured by the two methods is consistent. The error between the DPHP-DTS method and the TDR method is mainly reflected in the sand from 28 February to 4 June. At this time, the change rate of the sand *θ_v_* is small, and the TDR method is prone to generating errors under a low moisture content, resulting in variable fluctuation (Figure 9d). In comparison, the distribution of *θ_v_* measured by the DPHP-DTS method is more uniform and stable (Figure 9c). Therefore, the DPHP-DTS method can effectively and dynamically monitor the temporal and spatial distribution of *θ_v_* under various moisture conditions.

## 6. Conclusions

By using a new fitting algorithm, this study further improved the DPHP-DTS method, considering the bulk density calibration. Under different heating strategies, the thermal pulse processes of sand and loess were tested, and the performance of the improved DPHP-DTS method when applied to sand and loess was evaluated. Then, the *C_v_*, *ρ_b_*, and *θ_v_* measurement results were compared with the measurement results obtained by the DSC method, the steady-state plate method, the cutting ring method, and the oven drying method, which verified the effectiveness of the improved DPHP-DTS method. Finally, during the soil evaporation process, the temporal and spatial distribution of *θ_v_* in the sand and loess was measured by the DPHP-DTS method and compared with the monitoring results obtained by the TDR method. The specific conclusions are drawn as follows:

(1) When obtaining *C_v_* by the improved DPHP-DTS method under different heating strategies, the *RMSE_tem_* values of the *C_v_* determination are all smaller than the DTS temperature measurement accuracy of 0.1 K, which proves that the fitting algorithm can reduce the error caused by the DTS accuracy.

(2) With the increase in the heating duration and strength, the measurement accuracy of the improved DPHP-DTS method increases. The heating duration can be extended to improve the measurement accuracy under the low-power heating strategy.

(3) The percentage error between the *ρ_b_* measured by the DPHP-DTS method and the cutting ring method is less than 3%, which proves that the novel method is suitable for judging whether the in situ *ρ_b_* has changed or not.

(4) The results of the soil moisture measurements during the soil evaporation process show that the *RMSE_moi_* of loess is 0.008127 kg kg^−1^ and the *RMSE_moi_* of sand is 0.006425 kg kg^−1^. The improved DPHP-DTS method can describe soil evaporation in both sand and loess with good reliability and stability.

The DPHP-DTS method has the advantage of being distributed, and the temporal and spatial distribution of the moisture content in the soil can be obtained through dynamic monitoring. Therefore, the DPHP-DTS monitoring system has the potential to realize the real-time, large-scale, in situ monitoring of the moisture content of soil and provide a new means of researching topics such as soil evaporation, freeze-thaw cycle process, and surface heat balance. In addition, this paper only validated the improved DPHP-DTS method based on the laboratory test. Henceforth, the system should be further applied to in situ applications, and its performance in field monitoring should be evaluated.

## Figures and Tables

**Table 1:** Characteristics of the soil used.

	Sand	Loess
*ρ_b_* (g cm^−3^)	1.474	1.600
*ω_G_* (kg kg^−1^)	9.27%	19.77%
*c_S_* (J kg^−1^ K^−1^)	680	750
*λ* (W m^−1^ K^−1^)	1.163	1.244
*C_u_*	1.455	4.310
*C_c_*	0.960	0.690
*d*_50_ (mm)	0.750	0.019

**Table 2:** The strength and the duration of different heating strategies.

	Strength	20 W m^−1^	30 W m^−1^	40 W m^−1^
Duration				
50 s		L5	M5	H5
70 s		L7	M7	H7
120 s		L12	M12	H12

**Table 3:** Fitting results of *ρ_b_* at different locations in the sand and loess.

Height (cm)	Loess		Sand	
	*ρ_b_* (g cm^−3^)	R^2^	*ρ_b_* (g cm^−3^)	R^2^
6.5	1.602	0.9987	1.421	0.9723
12.5	1.626	0.9988	1.442	0.9972
20	1.641	0.9498	1.419	0.9965

## Data Availability

The datasets used for this study are available from the corresponding author upon reasonable request.

## Nomenclature

*C_v_*	soil volumetric heat capacity (J m^−3^ K^−1^)
*C_v_*_0_	volumetric heat capacity (J m^−3^ K^−1^) of the probe
*c_w_*	water specific heat capacity (J kg^−1^ K^−1^)
*c_s_*	soil solid specific heat capacity (J kg^−1^ K^−1^)
*C* *_u_*	uniformity coefficient
*C_c_*	curvature coefficient
* d *_ 50 _	mean grain size (mm)
∆*E*	difference between the molecular energy states driving Raman scattering (J)
*k*	Boltzmann constant (J K^−1^)
*K*_0_	second kind of 0-order modified Bessel function
*K*_1_	second kind of 1-order modified Bessel function
*p*	Laplace transformation variable
*q*	heating strength (W m^−1^) per unit length
*R*(*z*)	ratio between Stokes and anti-Stokes intensities
*r*	distance (m) between the heating probe and the temperature probe
*r_0_*	radius (m) of the probe
*T*	temperature (K)
*T*_0_	initial temperature (K)
Δ*T_max_*	maximum temperature (K) rise value
*t*	time (s)
*z*	distance (m) along the cable
∆α	differential attenuation of the backscattered Stokes and anti-Stokes intensities
*λ*	soil thermal conductivity (W m^−1^ K^−1^)
*θ_v_*	volumetric water content (m^3^ m^−3^)
*ρ_w_*	water density (g cm^−3^)
*ρ_b_*	soil bulk density (g cm^−3^)
*ω_G_*	mass water content (kg kg^−1^)
